# Anesthetic management of scapular Y-osteotomy using a combination of suprascapular nerve block and erector spinae plane block for Sprengel deformity associated with Klippel-Feil syndrome: a case report

**DOI:** 10.1186/s40981-023-00647-3

**Published:** 2023-08-29

**Authors:** Mizuho Okada, Nobuhiro Tanaka, Takanori Suzuka, Yuma Kadoya, Takashi Saisu, Masahiko Kawaguchi

**Affiliations:** 1 Department of Anesthesiology, Nara Medical University, 840 Shijo-Cho, Kashihara, Nara, 634-8522 Japan; 2 Chiba Child & Adult Orthopaedic Clinic, 3-24-2 Ayumino Minami, Chiba, Midori-ku 266-0033 Japan

**Keywords:** Peripheral nerve block, Suprascapular nerve block, Erector spinae plane block, Pediatric anesthesia

## Abstract

**Background:**

Klippel-Feil syndrome (KFS) occurs in 1/40,000 individuals and is characterized by cervical fusion. Thirty percent of patients with KFS present with Sprengel deformity, leading to orthopedic problems and limited shoulder abduction. No reports exist regarding anesthetic procedures for pediatric scapular osteotomies.

**Case presentation:**

We report a case of a 4-year-and-7-month-old boy (95.6 cm, 14.7 kg) who underwent left scapular osteotomy. At the age of 8 months, he also underwent a right lower lobectomy due to a congenital pulmonary airway malformation. We decided to use a combination of suprascapular nerve block (SSNB), erector spinae plane block (ESPB), and general anesthesia. He received regular acetaminophen administration and fentanyl 5–10 μg/hour intravenously until 20 h postoperatively and remained on ≤ 2/10 in the Wong-Baker Face Scale (0: no hurt; 10: hurts worst).

**Conclusion:**

The combination of SSNB and ESPB could be an option for perioperative analgesia for scapular osteotomies.

## Background

Klippel-Feil syndrome (KFS) is a congenital anomaly characterized by the triad of cervical vertebral body fusion, low posterior hairline, and short neck [1, 2]. Based on an epidemiological study of KFS, the prevalence of KFS in newborns worldwide is approximately 1/40,000 [3]. Sprengel deformity, associated with 30% of KFS, is a condition where the scapula is fixed in a cephalad-elevated position from birth [4]. Another report regarding Sprengel deformities indicated a frequency of 0.3 cases per 10,000 live births [5]. Patients commonly experience orthopedic problems related to the pterygoid neck and limited shoulder elevation due to the limited range of scapulothoracic joint motion. Despite that scapular osteotomy involves osteotomy and muscle dissection and is expected to be painful, no anesthetic reports exist regarding this procedure. Considering the innervation of the scapula and site of the surgical incision, we report a case of anesthetic management in which a combination of suprascapular nerve block (SSNB) and erector spinae plane block (ESPB) was used in addition to general anesthesia to obtain satisfactory analgesia.

## Case presentation

**Fig. 1:**
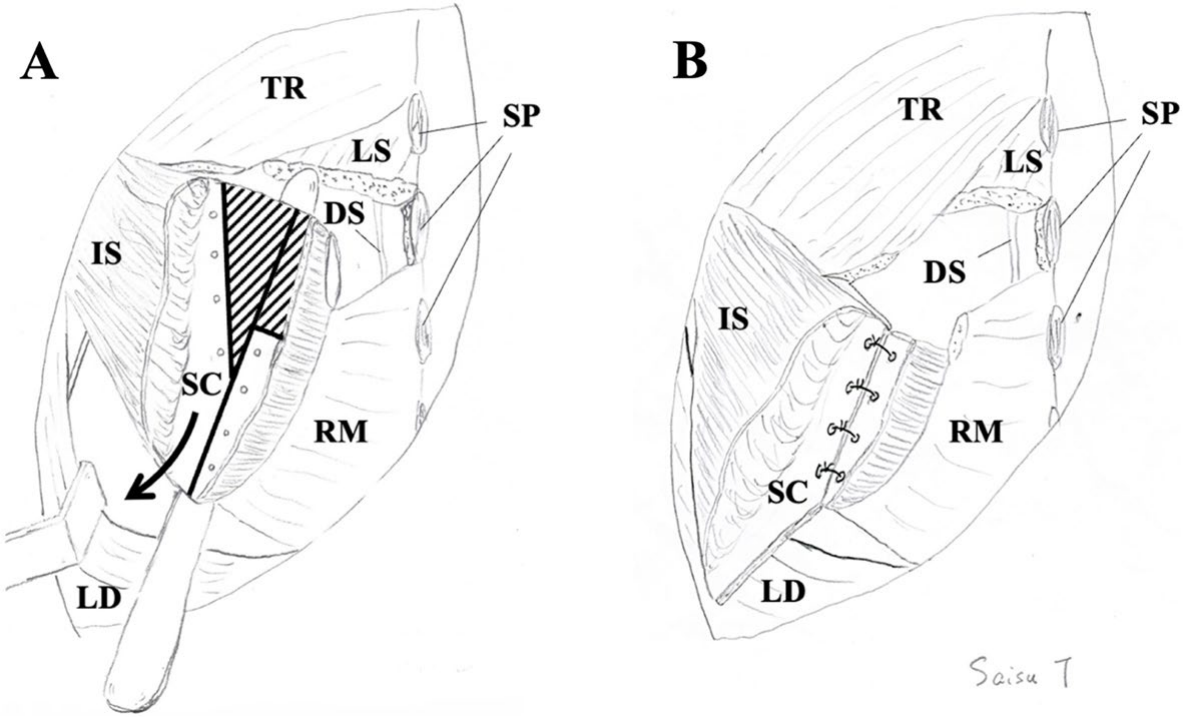
Surgical schematic of scapular Y-osteotomy proposed by Saisu (handwritten by himself). **A** Solid lines indicate the lines of osteotomies. Shaded lines indicate the areas to be resected. The lateral part of the scapula was pulled down while simultaneously improving downward rotation. The black arrow indicates the direction of the pull-down of the scapula. **B** After pulling down and suturing the lateral fragment of the scapula. The fascia of the infraspinatus was repaired, and the latissimus dorsi was sutured onto the infraspinatus after this process. DS, dorsal scapular nerve; IS, infraspinatus; LD, latissimus dorsi; LS, levator scapulae; RM, rhomboid major; SC, scapula; SP, spinous process; TR, trapezius

**Fig. 2:**
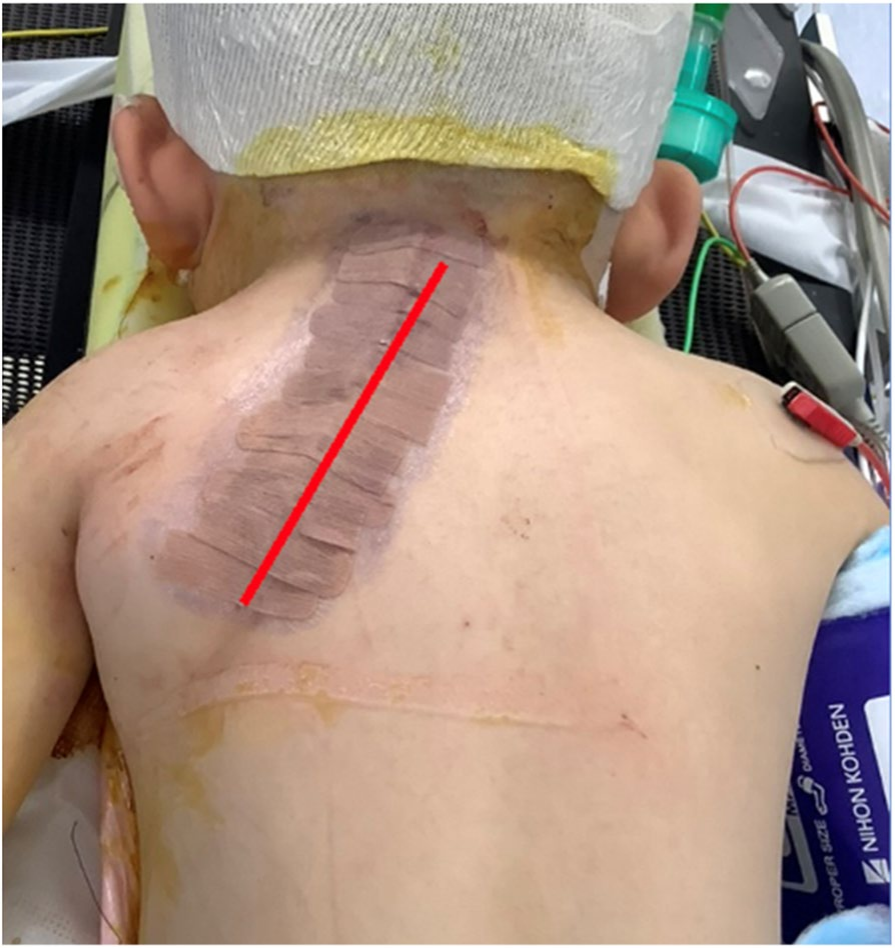
Line of skin incision in this case (red line)

A 4-year-and-7-month-old boy (95.6 cm, 14.7 kg) with Sprengel deformity associated with KFS underwent a left-sided scapular Y-osteotomy (Figs. 1 and 2). Symptoms of KFS included fusion of the cervical C2-3 vertebrae and C5-6 vertebrae. Previously, he underwent a right lower lobectomy at 8 months of age due to a congenital pulmonary airway malformation.

**Fig. 3:**
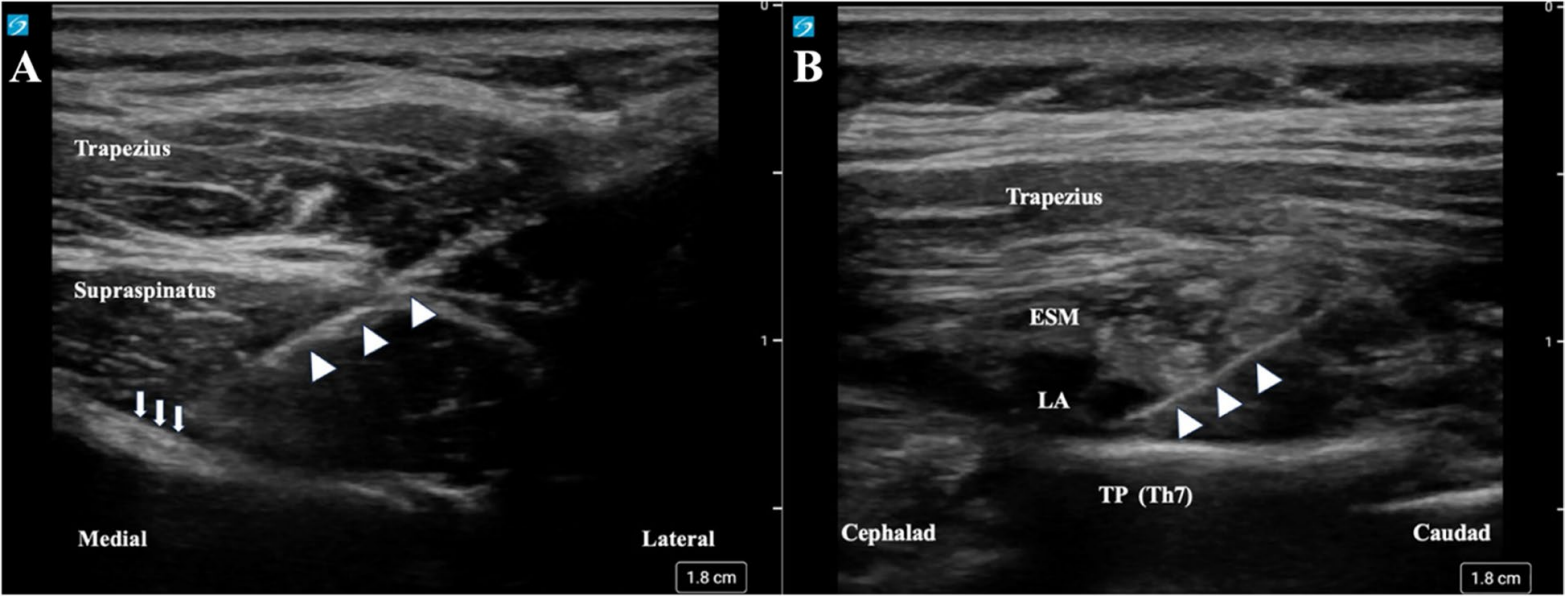
Ultrasound image of peripheral nerve block. **A** Ultrasound image of suprascapular nerve block. The needle (white arrowhead) is moved toward the suprascapular nerve (white arrow). **B** Ultrasound image of the erector spinae plane block. The needle (white arrowhead) is inserted from the caudad to the cephalad to avoid damaging the surgical site. ESM, erector spinae muscle; LA, local anesthetic; TP, transverse process

Slow induction was achieved using sevoflurane and nitrous oxide. After securing the peripheral venous line, fentanyl and rocuronium were administered, and the patient was intubated. Tracheal intubation was performed smoothly with McGRATH™ MAC (Covidien, Tokyo, Japan), considering the pterygoid neck and KFS. Anesthesia was maintained with sevoflurane and remifentanil at 0.1–0.2 μg/kg/min. Nitrous oxide was administered only during anesthesia induction. After induction, the SSNB was performed in the supine position. The needle was inserted using the in-plane technique under ultrasound guidance (Sonosite PX; Fujifilm Sonosite, Japan). Following negative blood aspiration, SSNB was performed using 5 mL of 0.25% ropivacaine (Fig. 3A). After the patient was placed in the prone position for surgery, ESPB was performed at the Th6–7 level with 10 mL of 0.2% ropivacaine (Fig. 3B). The decision regarding local anesthetic concentrations and amount for SSNB and ESPB was based on previous studies to ensure the administration of a maximum total amount of 2.5 mg/kg of ropivacaine [6, 7].

The operative and anesthetic times were 172 and 300 min, respectively. The patient received intravenous fentanyl (75 μg; 25 µg at intubation, at the start of the operation, and at the end of the operation, respectively) and 220 mg of acetaminophen. Postoperative pain was managed in the ward with regular acetaminophen administration (every 6 h) and continuous intravenous infusion of 10 μg/h fentanyl combined with 10 μg fentanyl for intravenous patient-controlled analgesia (IV-PCA) with a 10-min lockout to obtain reliable overnight analgesia. Pain was assessed using the Wong-Baker face scale (0: no hurt; 10: hurts worst) after surgery, which was 2, 2, and 0 at 2 h, 1 day, and 2 days postoperatively, respectively. IV-PCA was discontinued 22 h postoperatively because of nausea and the absence of pain at rest. The patient was discharged on postoperative day six without any adverse events.

## Discussion

Sprengel deformity has a rare clinical presentation; however, it is the most common congenital shoulder anomaly. It is commonly linked to various conditions, including KFS, scoliosis, and rib anomalies. Surgical management is typically warranted for severe cases, with the goal of improving cosmesis and function [8]. While reports on airway management for KFS and surgical techniques for Sprengel deformity are available, to our knowledge, no reports on the anesthetic methods of osteotomies exist for Sprengel deformity with KFS. Surgery for Sprengel deformity includes Green’s and Woodward’s procedures, which release the muscles attached to the scapula, and Wilkinson’s procedure, which mainly involves osteotomy; however, it is difficult to achieve satisfactory results with either of these techniques alone. In this case, a Y-osteotomy was selected to simultaneously lower the lateral part of the scapula and improve downward rotation at the same time [9].

Although there are a variety of surgical techniques, most attention is focused on outcomes related to range of motion and cosmetic aspects, not on pain and analgesia. The current trend in perioperative anesthesia management is a multimodal analgesic strategy that combines the regular administration of acetaminophen, peripheral nerve blocks, and opioids, as necessary.

We selected SSNB and ESPB as peripheral nerve blocks for the following reasons.

Interscalene brachial plexus nerve block (ISB) may provide scapular analgesia. The identification of the fifth and sixth cervical spinal cords is extremely difficult because KFS is characterized by cervical fusion. Furthermore, because a left scapular osteotomy was performed in this case, ISB resulted in left phrenic nerve palsy. However, because the right lung to be compensated for had undergone lobectomy, we decided not to perform ISB for respiratory function. The risk of pneumothorax was also considered, and a paravertebral block was not performed because of the post-lobectomy right lung.

The dorsal scapular nerve innervates the levator scapula, minor rhomboids, and major rhomboids. Intraoperative electrical stimulation of the dorsal scapular nerve by surgeons was scheduled to prevent nerve injury and identify the innervating muscles; therefore, we decided not to perform a dorsal scapular nerve block.

SSNB, which is expected to provide scapular analgesia and avoid phrenic palsy, can be performed using either a proximal or a distal approach [10, 11]. We decided to use the distal approach because of the difficulty in identifying the cervical cord due to cervical fusion, which is a characteristic of KFS.

We avoided paravertebral block but performed ESPB to avoid the risk of pneumothorax.

Although ESPB is considered clinically effective, the results of studies examining its mechanism (i.e., whether the local anesthetic diffuses into the paravertebral space) do not fully support the efficacy of ESPB, and opinions are divided [12]. ESPB is a nerve block of the posterior branch of the spinal nerve, which was exactly in line with the wound in this case.

In support of these choices, the remifentanil dose was temporarily increased to 0.2 μg/kg/min during the dissection of the levator scapulae, while maintaining stability in vital signs at 0.1 μg/kg/min in other instances.

The Wong-Baker face scale scores, in this case, varied below two. Regular administration of acetaminophen every 6 h and IV-PCA of fentanyl that continued until 22 h postoperatively may have contributed to the good scores. However, the fact that the scapula and upper arm were immobilized with a postoperative deltoid bandage and that almost no pain was caused by body movement was also thought to have contributed to the good scores.

To our knowledge, this is the first report of anesthetic management in pediatric scapular osteotomies for Sprengel deformity with KFS. This case demonstrates that the combined use of SSNB and ESPB leads to successful and effective postoperative analgesia during scapular Y-osteotomy.

## Data Availability

The data relevant to this case report are unavailable for public access because of patient privacy concerns.

## Abbreviations

KFS: Klippel-Feil syndrome
SSNB: Suprascapular nerve block
ESPB: Erector spinae plane block
IV-PCA: Intravenous patient-controlled analgesia
ISB: Interscalene brachial plexus nerve block
